# Lesion mimic gene *HLM1* positively regulates cell death and disease resistance in rice

**DOI:** 10.3389/fpls.2025.1669235

**Published:** 2025-11-05

**Authors:** Peiyun Zhang, Chaofeng Zong, Yujie Gu, Le Mei, Qianwen Ge, Pengcheng Liu, Bojun Ma, Xifeng Chen

**Affiliations:** ^1^ College of Life Sciences, Zhejiang Normal University, Jinhua, China; ^2^ China-Mozambique Belt and Road Joint Laboratory on Smart Agriculture, Zhejiang Normal University, Jinhua, China

**Keywords:** rice, lesion mimic mutant, *Hlm1*, programmed cell death, disease resistance

## Abstract

Lesion mimic mutants are important resources for deciphering the molecular mechanism of programmed cell death (PCD) and defense response in plants. Here, we isolated a new dominant lesion mimic mutant, *Hlm1*, which exhibited a hypersensitive response (HR) like phenotype with excessive accumulation of reactive oxygen species (ROS) and constitutive expression of pathogenesis-related (*PR*) genes, displayed a spontaneous cell death and enhanced resistance to rice bacterial blight pathogens as well. Genetic analysis indicates that the lesion mimic phenotype of *Hlm1* is controlled by a dominant allele, which is tightly linked to a T-DNA insertion in rice chromosome 2, and a candidate gene *OsNRAT1*, encoding the Nramp (natural resistance-associated macrophage protein) aluminum (Al) transporter, was found to be highly up-regulated in the *Hlm1* mutant. Loss-of-function of *OsNRAT1* gene by CRISPR/Cas9 in the *Hlm1* mutant decreased the lesion mimic phenotype. Interestingly, OsSPL1, a validated component of rice defense responses, was identified to interact with OsNRAT1. In addition, transcriptome analysis showed that the elevated expression of *OsNRAT1* in the *Hlm1* mutant promotes the plant-pathogen interaction pathway and MAPK signaling pathway to activate downstream defense genes, while simultaneously inducing the production of diterpenoid phytoalexins to combat pathogen invasion. Overall, our results will enhance the understanding of the molecular mechanisms underlying programmed cell death and disease resistance in rice.

## 1 Introduction

Plants have evolved various defense systems to protect themselves against pathogens with complex signaling pathways (Hofius et al., 2007). Hypersensitive response (HR), which is characterized by the rapid induction of local programmed cell death (PCD) around the infected cells, is one of the most efficient and immediate resistance reactions to defend against pathogens in plants (Lorrain et al., 2003). Upon perception of pathogen infection, the HR is rapidly activated at and around the infection site, as a strategy to restrict pathogen invasion or proliferation by triggering localized cell death (Singh et al., 2018). So far, it has been confirmed that several processes were associated with HR, such as the bursts of reactive oxygen species (ROS), the expression of pathogenesis-related (*PR*) genes, and the accumulation of phytoalexins (Shirsekar et al., 2014; Wang et al., 2017). However, the detailed molecular mechanism of HR is still poorly understood.

Plant lesion mimic mutants (*LMMs*), which spontaneously display HR-like cell death spots on leaves in the absence of pathogenic attack and usually display enhanced resistance to plant diseases by activating defense responses, are one of the most effective resources being used to unravel the mystery of HR (Shirsekar et al., 2014; Jin et al., 2015). Nowadays, the *LMMs* have been found in multiple plants, such as maize (*Zea mays*; Hoisington et al., 1982), *Arabidopsis* (Dietrich et al., 1994), rice (*Oryza sativa*; Takahashi et al., 1999) and barley (*Hordeum vulgare*; Wolter et al., 1993). To date, a large number of the *LMMs* have been identified from rice, and at least 61 genes have been cloned (Chen et al., 2025). The proteins encoded by those cloned genes mainly can be functionally classified into four types. Type I refers to gene transcription and protein translation, such as *SPL5* encodes a putative splicing factor 3b subunit 3 involved in pre-mRNA processing (Chen et al., 2009, 2012), and *SPL33* encodes a eukaryotic translation elongation factor 1 alpha (eEF1A)-like protein which functions in protein synthesis (Wang et al., 2017; Zhao et al., 2017). Type II refers to protein post-translational modifications (PTMs), such as *SPL11* encodes E3 ubiquitin ligases of the ubiquitination system (Ma et al., 2019) and *SPL50* encodes an ARM repeat protein, which plays a significant role in the ubiquitination processes by its ARM repeat domain (Ruan et al., 2024). Type III refers to intracellular vesicular trafficking, such as *SPL28* encodes clathrin-associated adaptor protein complex 1 subunit which is localized in the Golgi (Qiao et al., 2010), *OsSEC3a* and *RLS2* encode the exocyst complex subunits involved in exocytosis (Ma et al., 2018; Tu et al., 2015). Type IV refers to catalyzation of metabolism, such as *SPL29* encodes a UDP-N-acetylglucosamine pyrophosphorylase that modulates the level of uridine 5′-diphosphoglucose-glucose (UDPG) in plants, thereby regulating the accumulation of ROS and the development of the lesion mimic phenotype (Xiao et al., 2017), *OsPHD1* encoding a UDP-Glucose epimerase, causes jasmonic acid (JA) accumulation and enhanced bacterial blight resistance in rice (Gao et al., 2022). Due to the diversity of the proteins encoded by these *LMM* genes and their involvement in many aspects of regulation, the molecular mechanisms of HR-mediated cell death and defense response in plants seem to be extremely complicated.

Interestingly, most of the lesion mimic mutants have been identified so far are recessive mutants but rarely dominant. According to the genetic mode of the *LMM* genes cloned from rice, only 2 genes, *NH1* and *SPL18*, are completely dominant (Zhu et al., 2020; Sathe et al., 2019; Qiu et al., 2021). *NH1* is a homologous gene of *AtNPR1* in rice, and overexpression of *NH1* can constitutively activate immune response and induce HR-like cell death (Chern et al., 2005). The *Spl18* mutant was mutagenized by an activation-tagging T-DNA insertion in the promoter of *OsAT1* gene, of which the transcriptional level was significantly activated in the *Spl18* mutant (Mori et al., 2007). This rarity suggests that gain-of-function mutations in key immune regulators are often detrimental, but their study can reveal critical, dose-sensitive nodes in the HR signaling network. The random integration of an enhancer-containing T-DNA segment can activate the expression of adjacent genes, leading to gain-of-function phenotypes. This T-DNA tagging approach not only allows for the straightforward identification of these flanking genes but also provides a unique capacity to uncover genetically redundant genes, which are typically difficult to identify through standard mutagenesis methods. To further elucidate the molecular mechanism of the HR, we generated a population of T-DNA insertion transgenic rice plants. From this population, we identified a mutant, designated *HR-like lesion mimic 1* (*Hlm1*), which showed spontaneous HR-like cell death on the leaf blade and exhibited enhanced resistance to rice bacterial blight. Therefore, conducting genetic analysis and allele cloning of the *Hlm1* mutant to identify the target gene, followed by functional characterization of its encoded protein, will provide deeper insights into the role of *LMM* genes in regulating programmed cell death and disease resistance mechanisms. Studying this rare dominant mutant can reveal a novel mechanism controlling HR and disease resistance. This finding holds promise for breeding rice varieties with durable resistance.

## 2 Materials and methods

### 2.1 Plant materials and growth conditions

Rice lesion mimic mutant *Hlm1* was isolated from the T_0_ transgenic plants of Nipponbare (*Oryza sativa* ssp. *japonica*), which was transformed with a pCAMBIA1300 vector containing 2×CaMV35S promoter in the T-DNA fragment. By self-crossing, the homozygous *Hlm1* mutant from T_4_ generation was used for the following experiments. All plants were grown under normal conditions in summer field of South China. For genetic analysis and gene mapping, the *Hlm1* mutant was crossed with a rice variety 9311 (*Oryza sativa* ssp. *indica*) and the F_1_ plants were self-crossed to obtain the F_2_ population.

### 2.2 Histochemical staining

Rice plants were grown in the field till to two-month-old stage, the leaves of the *Hlm1* mutant without lesions and the wild-type (WT, Nipponbare) were collected and immediately stained for histochemical assay. All experiments repeated biological three times, with each replicate consisting of leaf samples from at least five different plants (n ≥ 5). Cell death in WT (negative control) and *Hlm1* mutant leaves was assessed by trypan blue (TPB; Coolaber, China). Leaves were stained in 2.5 mg·mL^−1^TPB for 10 minutes at room temperature, then destained by boiling in ethanol for 2–3 hours. The formation of blue spots was examined after destaining (Chen et al., 2012). Superoxide anion (O_2_^−^) accumulation in WT and *Hlm1* leaves was compared using Nitro-blue tetrazolium (NBT) staining (Jabs et al., 1996). Leaf samples were vacuum-infiltrated with 0.2 mg·mL^−1^ NBT solution (Aladdin, China) for 15 minutes and then incubated in darkness for 8 hours. Subsequently, the leaves were boiled in absolute ethanol for destaining. The ethanol was replaced several times until the leaves were fully decolorized. Finally, the formation of dark blue formazan spots was examined. Hydrogen peroxide (H_2_O_2_) accumulation was compared between WT and *Hlm1* leaves using diaminobenzidine (DAB) staining (Chen et al., 2012). Leaves were vacuum-infiltrated with 1 mg·mL^-1^ DAB solution (pH 3.8; Sigma, USA) and incubated in darkness for 8 hours. They were then decolorized by boiling in ethanol for 30 minutes, followed by clearing in fresh ethanol at room temperature until fully bleached. The formation of brown precipitate was finally documented.

### 2.3 Bacterial blight resistance assay

Nine strains of the *Xanthomonas oryzae* pv. *oryzae* (*Xoo*), POX71, PXO79, POX86, PXO87, PXO99, PXO112, POX124, PXO145 and PXO280, were obtained from Chinese Academy of Agricultural Sciences. The *Xoo* strains were cultured on the Potato Dextrose Agar (PDA) plate containing 20 g of sucrose, 5 g of peptone, 0.5 g Ca(NO_3_)_2_, 0.43 g Na_2_HPO_4_, and 0.05 g FeSO_4_ per liter, and incubated at 28°C for 2 days. The bacterial lawns were suspended in sterile distilled water and adjusted to an optical density of OD_600_ = 1.0 for plant inoculation. At two-month-old, rice plants of *Hlm1* mutant and WT control were inoculated with *Xoo* using the leaf-clipping method (Chen et al., 2021). Each bacterial strain was applied to 10 leaves, with three biological replicates. Lesion length was measured 14 days post-inoculation to assess disease resistance.

### 2.4 Aluminum tolerance evaluation

Rice seedlings (7-day-old) of the *Hlm1* mutant and the WT plants were exposed to 0.5 mM CaCl_2_ which containing 30, 50, or 100 μM Al (pH 4.5) for 24 h. Root lengths of the treated plants were measured with a ruler before and after Al tolerance treatments. The relative root elongation was calculated as (root elongation with Al)/(root elongation without Al)×100%. Every treatment with three independent replicates and six roots were measured for each treatment. For detecting the accumulation of Al in root tips, rice seedlings (3-day-old) of the *Hlm1* mutant and the WT plants planting in the solution containing 0.5 mM CaCl_2_ (pH 4.5) were treated with 50 μM AlCl_3_ for 24 h, and then staining with hematoxylin solution containing 2 mg/mL hematoxylin and 0.2 mg/mL KIO_3_ for 30 min (Tang et al., 2000). After washing off the excess hematoxylin on the surface of the samples, the root tips were photographed under stereomicroscope (OLYMPUS SZX10). Each treatment had three biological replicates, with at least five seedlings per replicate.

### 2.5 Agronomic traits analysis

At the mature stage, ten individual plants of the *Hlm1* mutant and the WT control were randomly selected to measure the agronomic traits of plant height, tiller number, flag leaf length and width, panicle length, grain number per panicle, seed setting ratio and 1000-grain weight according to the standard evaluation system (Xuan et al., 2019). The specific measurements were defined as follows: Plant height was the vertical distance from the ground to the tip of the tallest panicle (excluding the awn). Tiller number was the count of the main stem and all effective tillers bearing a panicle. Flag leaf length and width were measured on the main stem flag leaf. Panicle length was the distance from the panicle base to its tip (excluding the awn). Grain number per panicle was the total count of all grains on a panicle. Seed-setting rate was the percentage of filled grains among the total grains, calculated from five random panicles. Thousand-grain weight was the average weight of three sets of 1000 filled grains.

### 2.6 TAIL-PCR and verification PCR

The genomic DNA of rice leaves was isolated by the common CTAB extraction method. Three specific primers (RB1 to RB3) on T-DNA right border and three arbitrary degenerate primers (**Supplementary Table S3**) were used for TAIL-PCR according to the method of Liu et al (Liu et al., 1995). The products of tertiary PCR were cloned into the pMD18-T Vector (TaKaRa, Japan) following the manufacturer’s instructions, and then sequenced to get the sequences of the insertion fragments. To verify the insertion site of T-DNA, three primers (P1, P2 and RB3; **Supplementary Table S3**) were mixed equally for PCR amplification using GoTaq Master Mix (Promega, USA). PCR process was performed as: (1) 94°C for 5 min; (2) 94°C for 30 s, 58°C for 30 s and 72°C 60 s, 35 cycles; (3) 72°C for 5 min. PCR products were examined via 1% agarose-gel electrophoresis and stained with ethidium bromide (EB).

### 2.7 Gene expression analysis by RT-qPCR

At the beginning of heading stage, when the lesion mimic spots of the *Hlm1* mutant first appeared, leaf samples were collected from the *Hlm1* mutant and WT control plants. The experiment repeated biological three times, each replicate pooled leaf tissue from at least five individual plants. Total RNAs of the leaves of the *Hlm1* mutant and WT were extracted using RNeasy Plant Mini Kit (QIAGEN, Germany) and RNase-Free DNase Set (QIAGEN) following the manufacturer’s instructions. Synthesis of first-strand cDNAs from RNA was carried out by the kit of M-MLV Reverse Transcriptase (Promega, USA) according to the manufacturer’s instruction. Fast SYBR Green Master Mix reagent (Applied Biosystems, USA) was used for real-time PCR. The thermal cycle was performed on the Step One Real-Time PCR System (Applied Biosystems) following: 95°C for 20 s; 40 cycles of 95°C for 3 s and 60°C for 30 s. A rice housekeeping gene *Actin* was used as the standardization control and the relative expression level of each gene was analyzed by 2^−ΔΔCt^ method (Livak and Schmittgen, 2001). Standard errors were calculated based on a minimum of three biological replicates. Gene specific primers are listed in **Supplementary Table S3**.

### 2.8 Gene editing mediated by CRISPR/Cas9

A single guide RNA (sgRNA) sequence of the *OsNRAT1* gene, 5’-AGGGACTGGTGAGATGAGAGAGG-3’, was designed by CRISPR Design program (http://crispr.mit.edu, October 20, 2020) based on the coding sequence of *OsNRAT1* gene. The bases underlined represent the PAM sequence. The DNA sequence of this sgRNA was cloned into the CRISPR/Cas9 vector pCAMBIA1300-pYAO-cas9. The verified construct was transformed into the *Hlm1* mutant using the *Agrobacterium*-mediated transformation with the method described by Nishimura et al (Nishimura et al., 2006). The genomic editing site of the T_0_ transgenic plants was amplified by PCR and sequenced with the primers named as Target F and Target R, respectively (**Supplementary Table S3**).

### 2.9 Construction of rice cDNA library

Total RNA was extracted from rice leaves of Nipponbare using the RNeasy Plant Mini Kit (Qiagen) and was purified with the RNase-Free Dnase Set (Qiagen) in accordance with the manufacturer’s instructions. Synthesis of first-strand cDNAs from the extracted RNA was performed using the M-MLV Reverse Transcriptase Kit (Promega) according to the manufacturer’s instructions. The double-stranded cDNA, obtained by PCR amplification, was ligated to the pGADT7 vector utilizing the In-Fusion HD Cloning Kit (TaKaRa). The resulting constructs were transformed into *Escherichia coli* DH5α for plasmid library amplification. Finally, the library plasmids were introduced into Y187 yeast competent cells using the LiAc transformation method (Gietz and Schiestl, 2007), plated on synthetic dropout medium (SD/-Leu) to select positive clones which were collected to establish the rice cDNA library.

### 2.10 Interacting protein screen by yeast two-hybrid system

The coding DNA sequence (CDS) of *OsNRAT1* were amplified using the PrimeSTAR HS DNA Polymerase Kit (TaKaRa, SA) with gene-specific primers (**Supplementary Table S3**). Subsequently, the CDS fragment of *OsNRAT1* was inserted into the pGBDT7 bait vector, utilizing the In-Fusion HD Cloning Kit (TaKaRa). The bait plasmid pGBKT7-*OsNRAT1* was transformed into Y2H yeast strain using the LiAc transformation method (Gietz and Schiestl, 2007). Through yeast two-hybrid mating assay (Letteboer and Roepman, 2008), the yeast strain containing the rice cDNA library was mated with the strain harboring the bait vector pGBKT7-OsNRAT1 to generate diploid yeast cells, which were subsequently selected on SD/-Leu/-Trp/-His/-Ade medium, 30°C. After 5–7 days, positive clones were then streaked onto SD/-Leu-Trp-His-Ade plates supplemented with X-α-Gal and incubated until blue colonies appeared. Blue colonies were subjected to colony PCR using pGADT7 universal primers AD and T7 (**Supplementary Table S3**). PCR products were analyzed by agarose gel electrophoresis and sequencing. The obtained sequences were analyzed by Blast (https://blast.ncbi.nlm.nih.gov) through the mRNA database to identify the corresponding proteins.

### 2.11 Proteins interaction verifying by Y2H

The CDS of *OsSPL1* was amplified using the PrimeSTAR HS DNA Polymerase Kit (TaKaRa, SA) with gene-specific primers (**Supplementary Table S3**). Subsequently, the CDS fragment of OsSPL1 was inserted into the pGADT7 prey vector, utilizing the In-Fusion HD Cloning Kit (TaKaRa). The vectors expressing BD-*OsNRAT1* and AD-OsSPL1 fused protein were co-transformed into Y2H Gold yeast competent cells. The proteins interaction was verified by examining the growth of yeast strains on nutrient-deficient media including SD/-Leu-Trp, SD/-Leu-Trp-His-Ade, and SD/-Leu-Trp-His-Ade supplemented with X-α-Gal. All culture plates were incubated at 30°C for 3 to 5 days, after which colony growth and color development were observed and recorded.

### 2.12 Bimolecular fluorescence complementation assay

The CDSs of *OsNRAT1* and *OsSPL1* were amplified using the PrimeSTAR HS DNA Polymerase Kit (TaKaRa) with gene-specific primers (**Supplementary Table S3**). Subsequently, the CDS of *OsNRAT1* was inserted into the YNE vector and fused with the N-terminal fragment of yellow fluorescent protein (YFP), with the recombinant vector expressing the YNE-OsNRAT1 fusion protein, while the CDS of *OsSPL1* was inserted into the YCE vector and fused with the C-terminal fragment of YFP, with the recombinant vector expressing the YCE-OsSPL1 fusion protein. The recombinant vectors YNE-OsNRAT1 and YCE-OsSPL1, along with control vectors YNE and YCE, were transformed into the competent cells of *Agrobacterium tumefaciens* strain GV3101. A single bacterial colony was cultured in LB medium with antibiotics to the logarithmic phase. The cells were then harvested and resuspended in infiltration buffer (10 mM MES, 10 mM MgCl_2_, 200 μM acetosyringone, pH 5.6), adjusting the final concentration to OD_600_ = 0.8. The bacterial suspensions were mixed in equal volumes according to the combinations of YNE-OsNRAT1 and YCE-OsSPL1, YNE-OsNRAT1 and YCE, YNE and YCE-OsSPL1. The mixtures were co-infiltrated into tobacco (*Nicotiana benthamiana*) leaves using a needleless syringe. For each experiment, at least two leaves from five independent tobacco plants were infiltrated. After infiltration, plants were kept in a growth chamber (16h light/8h dark, 23°C) for 48–72 hours. YFP fluorescence in the infiltrated areas was then observed using confocal microscopy (excitation 514 nm).

### 2.13 RNA-seq analysis

Two month after sowed, leaves were collected from the *Hlm1* mutant and WT control. Each group had three biological replicates, with each replicate comprising a pooled sample of leaves from ten individual plants. These samples were sent to the Beijing Genomics Institute (BGI) for RNA-seq analysis on an Illumina HiSeq2500 system. Raw reads were first processed to remove the low-quality reads and the adaptor sequences by SOAPnuke (Kawahara et al., 2013). The cleaning reads were mapped to the rice reference genome of Nipponbare (http://rice.plantbiology.msu.edu/) by Bowtie2 (Langmead and Salzberg, 2012), and the transcriptional level of genes were calculated by RSEM (Li and Dewey, 2011). Differentially expressed genes (DEGs) between the *Hlm1* mutant and the WT control were identified by DEGseq (Wang et al., 2010) with the Log_2_ (*Hlm1*/WT FRKM) ≥ 1 or ≤ -1 and the significance of adjusted *P* < 0.05. Enrichment analysis of the KEGG pathway of DEGs were performed on the web site of BGI (https://report.bgi.com). Heatmap analysis was performed using log_2_(*Hlm1*/WT FPKM) values for the genes listed in **Supplementary Table S4**.

## 3 Results

### 3.1 The *Hlm1* mutant displays HR-like lesion mimic phenotype and enhances disease resistance

**Figure 1:**
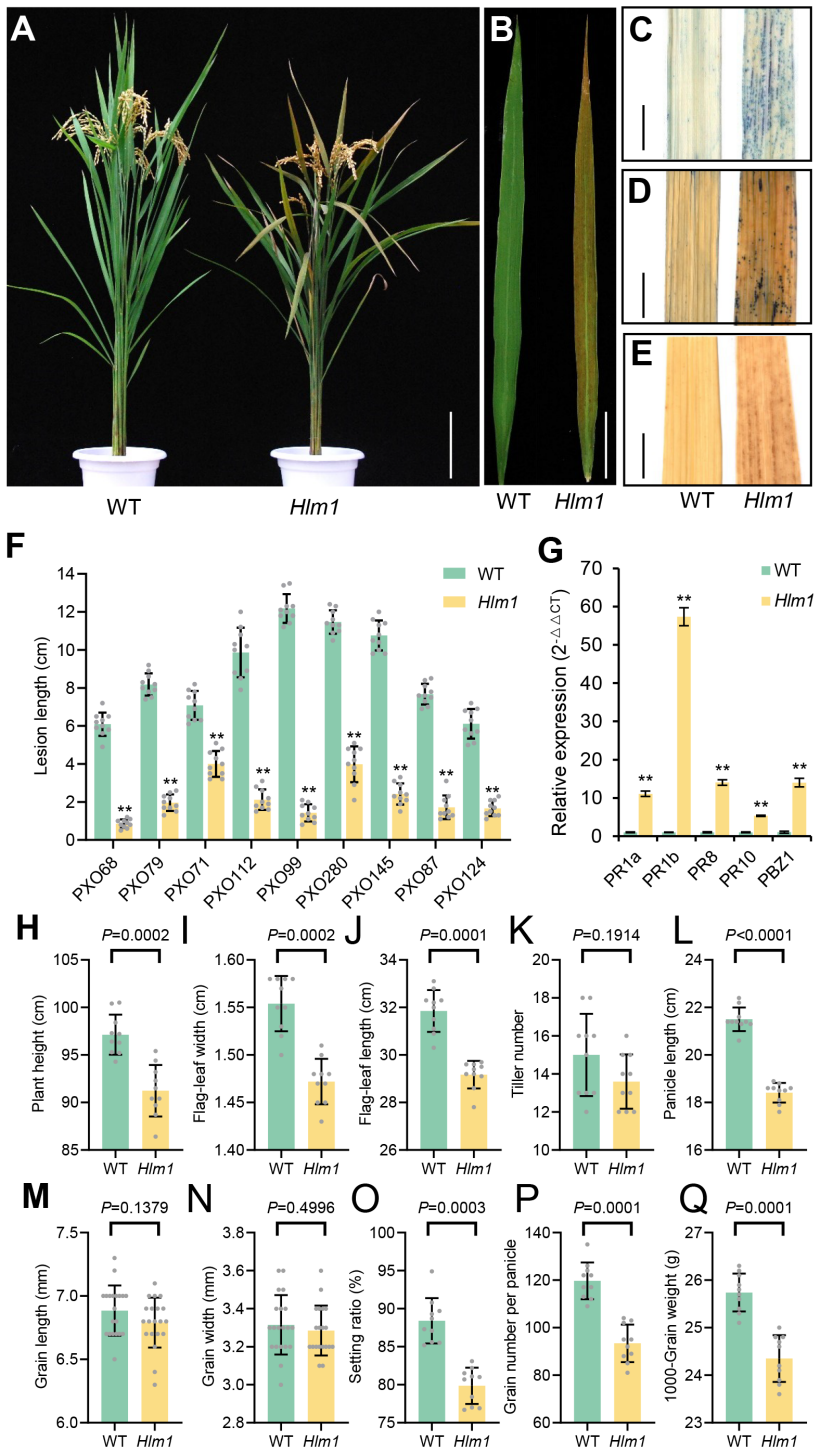
Phenotype of the *Hlm1* mutant. **(A)** Plants and **(B)** leaves of the *Hlm1* mutant and the WT control at the mature stage. **(C)** Trypan blue, **(D)** NBT and **(E)** DAB staining for the assays of cell death, accumulation of H_2_O_2_ and O_2_^−^, respectively. **(F)** Lesion length on the leaves infected with *Xoo* strains and measured 2 weeks after inoculation. **(G)** Expression of the pathogen related (*PR*) genes by RT-qPCR. **(H–Q)** agronomic traits of plant height, flag-leaf width, flag-leaf length, tiller number, panicle length, grain length, grain width, setting ratio, grain number per panicle and 1000-grain weight at the mature stage. Scale of bars are 20 cm in **(A)**, 5 cm in **(B)** and 1 cm in **(C–E)** Statistical analysis was performed with *t*-test and significant difference (*P* < 0.01) between the *Hlm1* mutant and the WT control was marked with ** above the bars.

In summer field, the rice lesion mimic mutant *Hlm1* spontaneously display small necrotic spots on the leaves at the heading stage in the absence of pathogen attack, and finally these necrotic spots scattered over the whole leaves at the mature stage (**Figures 1A, B**). Leaves without necrotic spots from the *Hlm1* mutant were analyzed by histochemical staining compared to WT. Trypan-blue staining showed that cells death was detected around the spots on the leaves of the *Hlm1* mutant, which was not found in the leaves of the WT control (**Figure 1C**); NBT and DAB staining showed that the over accumulation of ROS (O_2_^−^ and H_2_O_2_) in the *Hlm1* mutant (**Figures 1D, E**). These results indicated that the *Hlm1* mutant displayed programmed-cell death, which was similarly to the HR phenotype caused by oxidative burst in defense response. Additionally, the *Hlm1* mutant displayed enhanced and a broad-spectrum disease resistance to all evaluated rice bacterial blight strains (**Figure 1F**), and the defense-response marker genes *PR1a*, *PR1b*, *PR8*, *PR10* and *PBZ1* (Mei et al., 2006; Van-Loon et al., 2006) were transcriptionally induced in the *Hlm1* mutant (**Figure 1G**). The above results suggested that the defense response was constitutively activated in the *Hlm1* mutant. Therefore, the growth and development of the *Hlm1* mutant were delayed, so its main agronomic traits, such as plant height, flag-leaf length and width, panicle length, grain length, setting ratio, grain number per panicle and 1000-grain weight, were all significantly decreased when compared to the WT control (**Figures 1H, Q**).

### 3.2 Gene mapping reveals a candidate gene *OsNRAT1* activated by a T-DNA in *Hlm1* mutant

**Figure 2:**
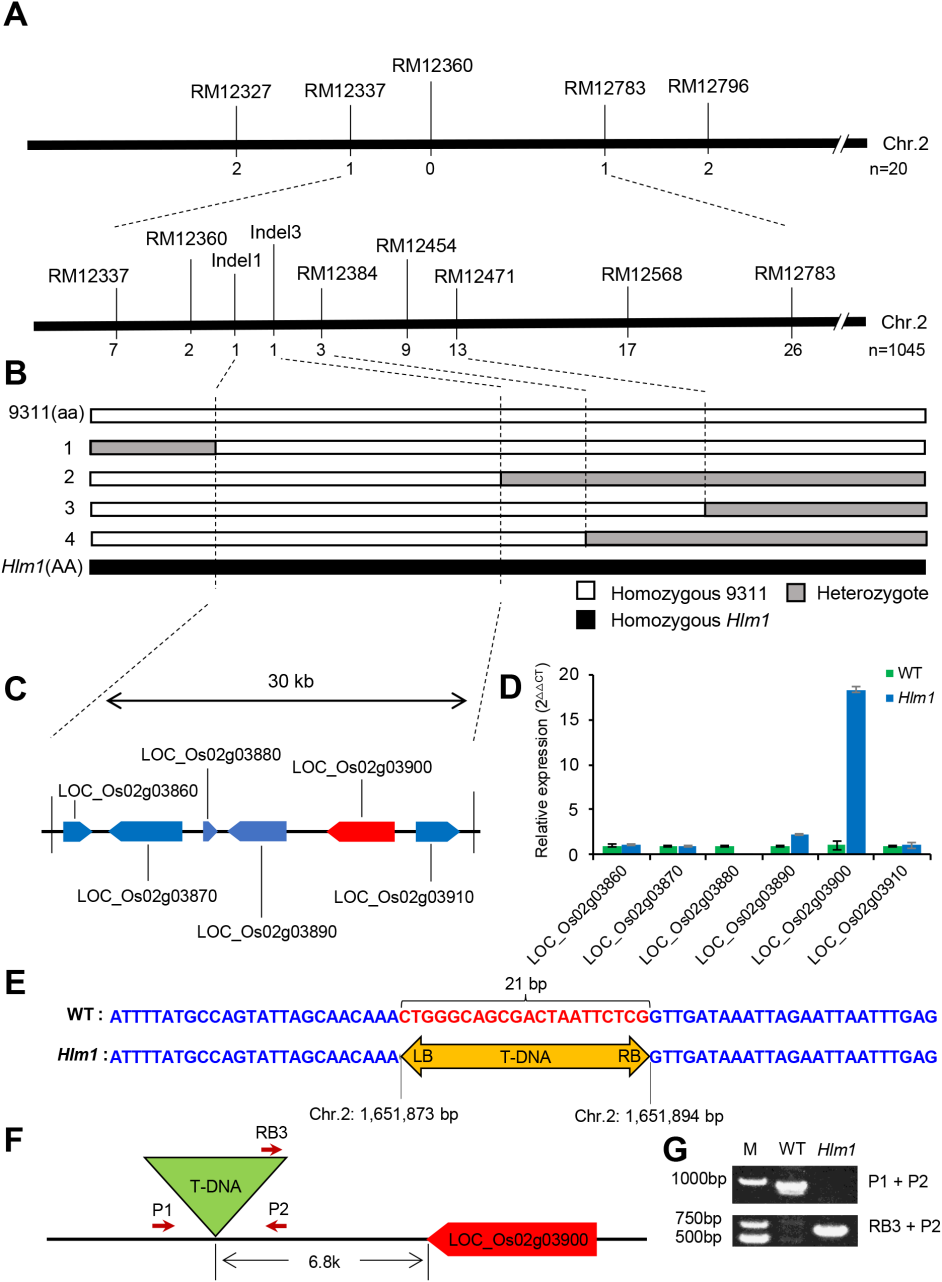
Genetic mapping of *HLM1*, verification of the T-DNA insertion site and expression assay of candidate genes. **(A)** Genetic map of the *Hlm1* locus on rice chromosome 2. Totally 20 and 1045 WT phenotype F_2_ individuals were used for primary and fine mapping, and the numbers under each marker represent the recombinants detected by the corresponding marker. **(B)** A graphical representation of recombinant genotypes. AA and aa represent the parental genotypes, and 1–4 denote selected wild-type recombinant F_2_ plants. **(C)** The candidate genes in the mapping region. **(D)** Relative expression of candidate genes by qRT-PCR. Data were shown as mean (three independent replicates) ± SD. **(E)** Location of the T-DNA insertion in rice chromosome 2 identified by the TAIL-PCR based sequencing. A 21-bp fragment shown with red color was replaced by the T-DNA after its insertion. The yellow box with double arrows represents the T-DNA, LB and RB means the left and right border. **(F)** Diagram of the T-DNA insertion site. P1, P2, and RB are primers designed to verify the T-DNA insertion site, with the arrow direction indicating the primer extension orientation. **(G)** Agarose-gel electrophoresis of PCR products amplified from the genomic DNAs of the *Hlm1* mutant and WT using the primers show in **(F)**. The lane marked “M” represents the DNA molecular weight marker.

Map-based cloning was used to isolate the *Hlm1* gene. A population of 4,280 F_2_ plants was derived from a cross between the *Hlm1* mutant and an *indica* rice variety 9311. Among them, there were 3,235 plants displaying lesion mimic and the remaining 1,045 plants were WT phenotype, and the segregation ratio was near to 3:1 by Chi-square test (*P* > 0.05), suggesting that *Hlm1* is a single dominant allele. Using 20 individual plants of the WT phenotype from F_2_ population, the *Hlm1* locus was primarily mapped between molecular markers RM12337 and RM12783 on rice Chromosome 2 (**Figure 2A**). Then, 1,045 WT phenotype F_2_ individual plants were analyzed and the *Hlm1* locus was mapped into a 30-kb region between the DNA markers Indel1 and Indel3 (**Figure 2A**). According to the rice genome annotation of Nipponbare (http://rice.plantbiology.msu.edu/), there are six genes in this mapping region (**Figure 2B**), whose expressions were analyzed by RT-qPCR (**Figure 2C**). Among them, only one gene (*LOC_Os02g03900*) was highly up-regulated in the *Hlm1* mutant and its expression level was 15 times more than that of WT, but the expressions of other genes were not or slightly changed.

Since the *Hlm1* mutant was identified from the transgenic rice plants, it was likely that the expression of *LOC_Os02g03900* was activated by an inserted T-DNA with a CaMV35S promoter. To validate the hypothesis, the TAIL-PCR was used to amplify the flanking sequence of the T-DNA right border from the *Hlm1* mutant, and found a T-DNA just inserted in the *Hlm1*-gene mapping region from 1,651,873 bp to 1,651,894 bp on rice Chromosome 2, in which a 21-bp fragment lost, based on the reference genome of Nipponbare (**Figure 2D**). According to the sequencing result, this T-DNA was 6.8-kp away from *LOC_Os02g03900* (**Figure 2E**). For verification of the insertion, two primers (P1 and P2) flanking the T-DNA and one primer (RB) on the T-DNA right border were designed (**Figure 2E**), and those three primers were mixed equally to do PCR for identification of the T-DNA insertion. A target fragment by the primers pair of RB and P2 was successfully amplified from the *Hlm1* mutant, and not from WT (**Figure 2G**). By contrast, the target fragment by the primer pair of P1 and P2 was amplified from WT, but not from the *Hlm1* mutant (**Figure 2F**).

**Figure 3:**
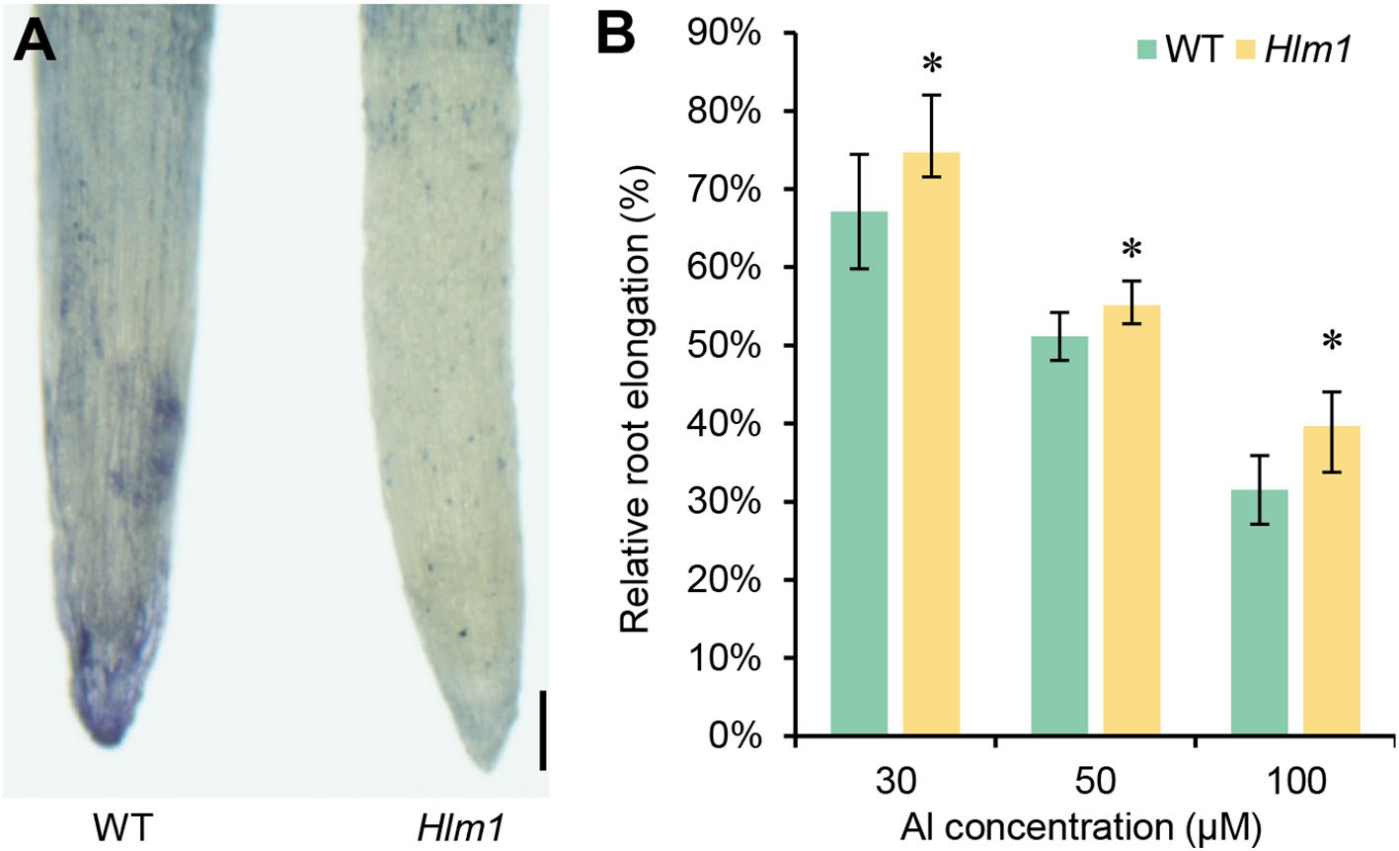
Aluminum (Al) tolerance of the *Hlm1* mutant. **(A)** Hematoxylin staining result in root tips of the *Hlm1* mutants and WT after 24 h exposure to 50 μM AlCl_3_, bar=2mm. **(B)** Al tolerance ability test of the *Hlm1* mutant and WT with different concentration of Al treatment. Data were shown as mean (three independent replicates) ± SD. Statistical analysis was performed by *t*-test, and * refers to a significant difference (*P* < 0.05) between the *Hlm1* mutant and WT.

Additionally, gene *LOC_Os02g03900* encodes the Nramp aluminum transporter 1 (OsNRAT1), has been reported to play a positive role in rice aluminum (Al) tolerance (Li et al., 2014). The tolerance ability of the *Hlm1* mutant to Al stress was also analyzed by the hematoxylin stain (**Figure 3A**). Compared to WT, the root tips of the *Hlm1* mutant displayed lighter staining in the presence of hematoxylin, indicating that the *Hlm1* mutant accumulated less Al. As shown in **Figure 3B**, the root elongation of the *Hlm1* mutant were less inhibited when compared to WT. The above results demonstrate that the *Hlm1* mutants increased tolerance to Al. Based on the above results, therefore, *LOC_Os02g03900* was considered as the candidate gene of *Hlm1*.

### 3.3 Knockout of *OsNRAT1* in *Hlm1* mutant suppresses the lesion mimic phenotype

**Figure 4:**
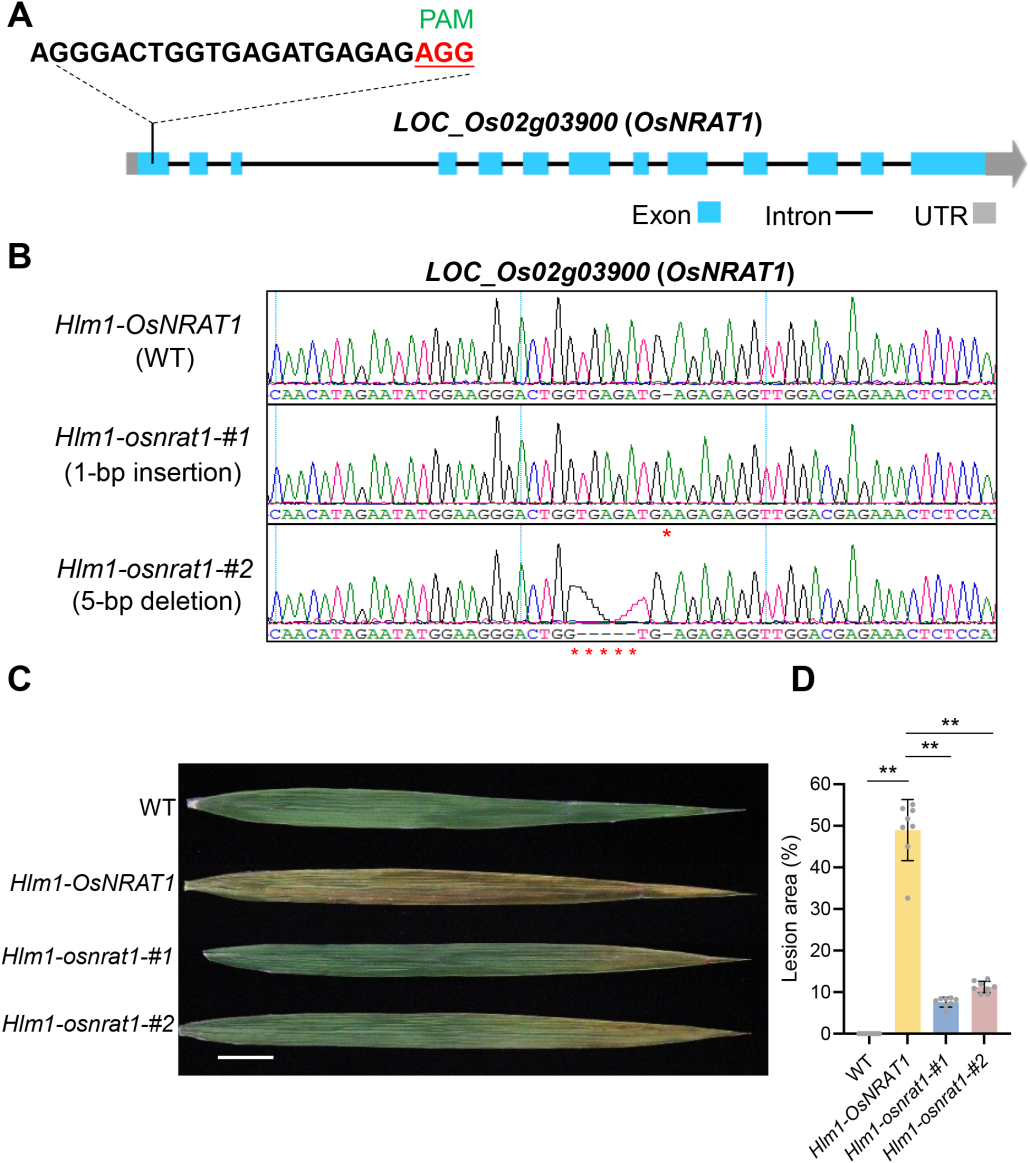
Knockout of *OsNRAT1* in rice *Hlm1* mutant and phenotype identification. **(A)** Target sequence and location of the single guide RNA (sgRNA) for CRISPR/Cas9 gene editing in *OsNRAT1*. The bases with red represent the PAM sequence, exons, introns and UTRs were marked with blue, black and grey, respectively. **(B)** Sequencing results of the target site in different *Hlm1* transgenic lines, *Hlm1-osnrat1-#1* (1-bp insertion) and *Hlm1-osnrat1-#2* (5-bp deletion), respectively. **(C)** Phenotype of the leaves collected from plants. Bar=2cm. **(D)** Lesion area on leaves of *Hlm1* mutants and *Hlm1* knockout lines. Leaf lesion area was quantified across eight individual plants from different lines. Statistical analysis by t-test revealed significant differences (P < 0.01) between the *Hlm1* mutant and the wild-type control, as well as between the *Hlm1* mutant and the two knockout lines. These significant differences are indicated with “**” above the corresponding bars in the histogram.

Genome editing mediated knockout of *OsNRAT1* (*LOC_Os02g03900*) by CRIPSR/Cas9 was carried out in the *Hlm1* mutant. A single guide RNA (sgRNA) was designed to target the first exon of *OsNRAT1* gene (**Figure 4A**), and the CRIPSR/Cas9 vector with the sgRNA was transformed into the *Hlm1* mutant. Two different knockout lines of *OsNRAT1* in the *Hlm1* mutant background, named *Hlm1-osnrat1-#1* (1-bp insertion) and *Hlm1-osnrat1-#2* (5-bp deletion), were identified from the transgenic plants (**Figure 4B**). Subsequently, the phenotypes of the two knockout lines *Hlm1-osnrat1-#1* and *Hlm1-osnrat1-#2* were identified in summer field. Compared to the WT, the *Hlm1* mutant displayed severe lesion mimic on leaves, while the knockout lines only showed slightly lesion mimic on the tip of leaves (**Figure 4C**). The results indicated that knockout of *OsNRAT1* could significantly suppress the lesion mimic phenotype of the *Hlm1* mutant.

### 3.4 OsNRAT1 interacts with OsSPL1 in yeast cells and tobacco leaves

**Figure 5:**
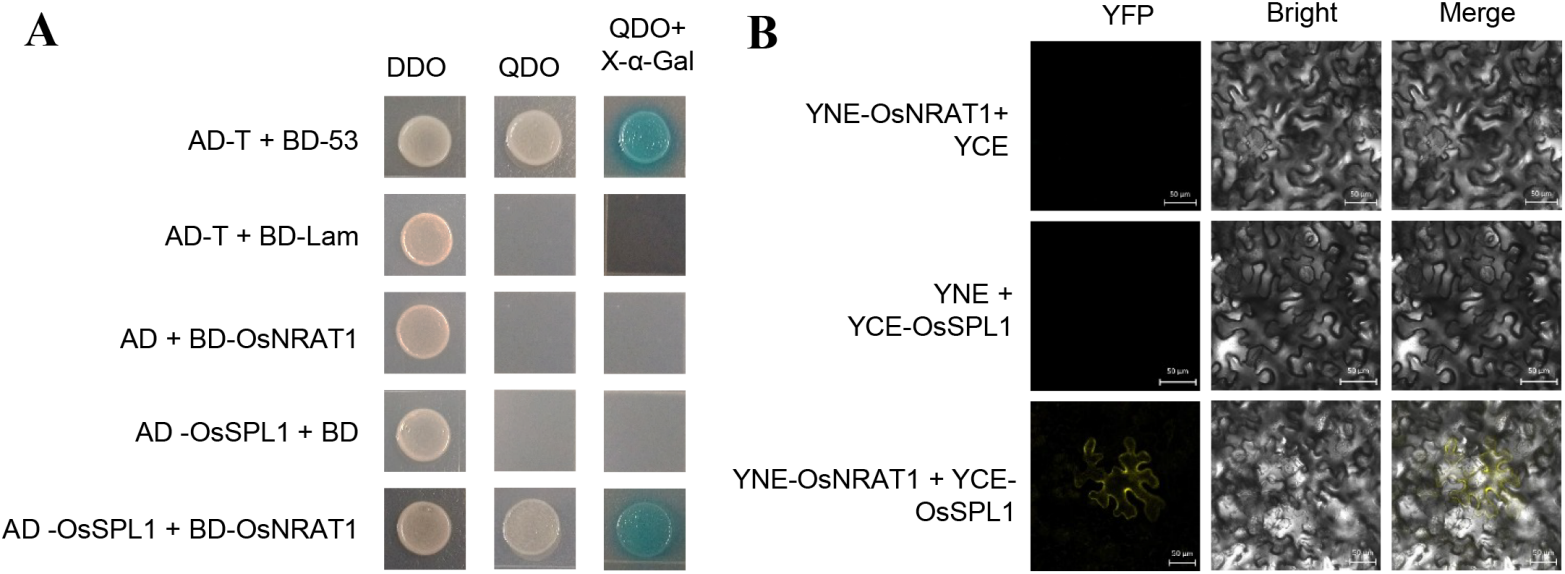
Protein interaction between OsNRAT1 and OsSPL1. **(A)** Yeast two-hybrid verification assay between OsNRAT1 and OsSPL1. The combinations AD-T with BD-53 serve as the positive control, while AD-T with BD-Lam constitute the negative control. The abbreviations DDO, QDO, and QDO + X-α-Gal represent distinct yeast synthetic dropout media: SD/-Leu-Trp, SD/-Leu-Trp-His-Ade, and SD/-Leu-Trp-His-Ade+X-α-Gal, respectively. **(B)** Bimolecular fluorescent complementary assay of the interaction between OsNRAT1 and OsSPL. The fused proteins, YCE-OsSPL1 and YNE-OsNRAT1, were co-expressed, and the fluorescence was observed after 48h in tobacco cells. YCE-OsSPL + YNE (empty vector) and YNE-OsNRAT1 + YCE (empty vector) were used for controls. Scale bar was 50 μm.

Since OsNRAT1 was reported to be an Al transporter (Li et al., 2014), how did it regulate the PCD and disease resistance in rice? To reveal the molecular mechanism of OsNRAT1, the Y2H technology was used to screen the OsNRAT1-interaction proteins from rice cDNA library. A total of 20 potential interacting proteins were ultimately identified, including OsSPL1 (sphingosine-1-phosphate lyase), which has been demonstrated to be associated with PCD and disease resistance in rice (Zhang et al., 2014). The direct interaction between OsSPL1 and OsNRAT1 was confirmed by Y2H, in which the full-length CDS of *OsSPL1* was used (**Figure 5A**). This interaction was further validated in tobacco leaf cells through bimolecular fluorescence complementation (BiFC) assay, revealing detectable fluorescence signals on the plasma membrane and cytoplasm (**Figure 5B**). Based on the results, it has been conclusively demonstrated that OsNRAT1 interacts with OsSPL1 at protein level and that they co-localize at both the plasma membrane and cytoplasm.

### 3.5 HR-like signaling pathways of the *Hlm1* mutant

**Figure 6:**
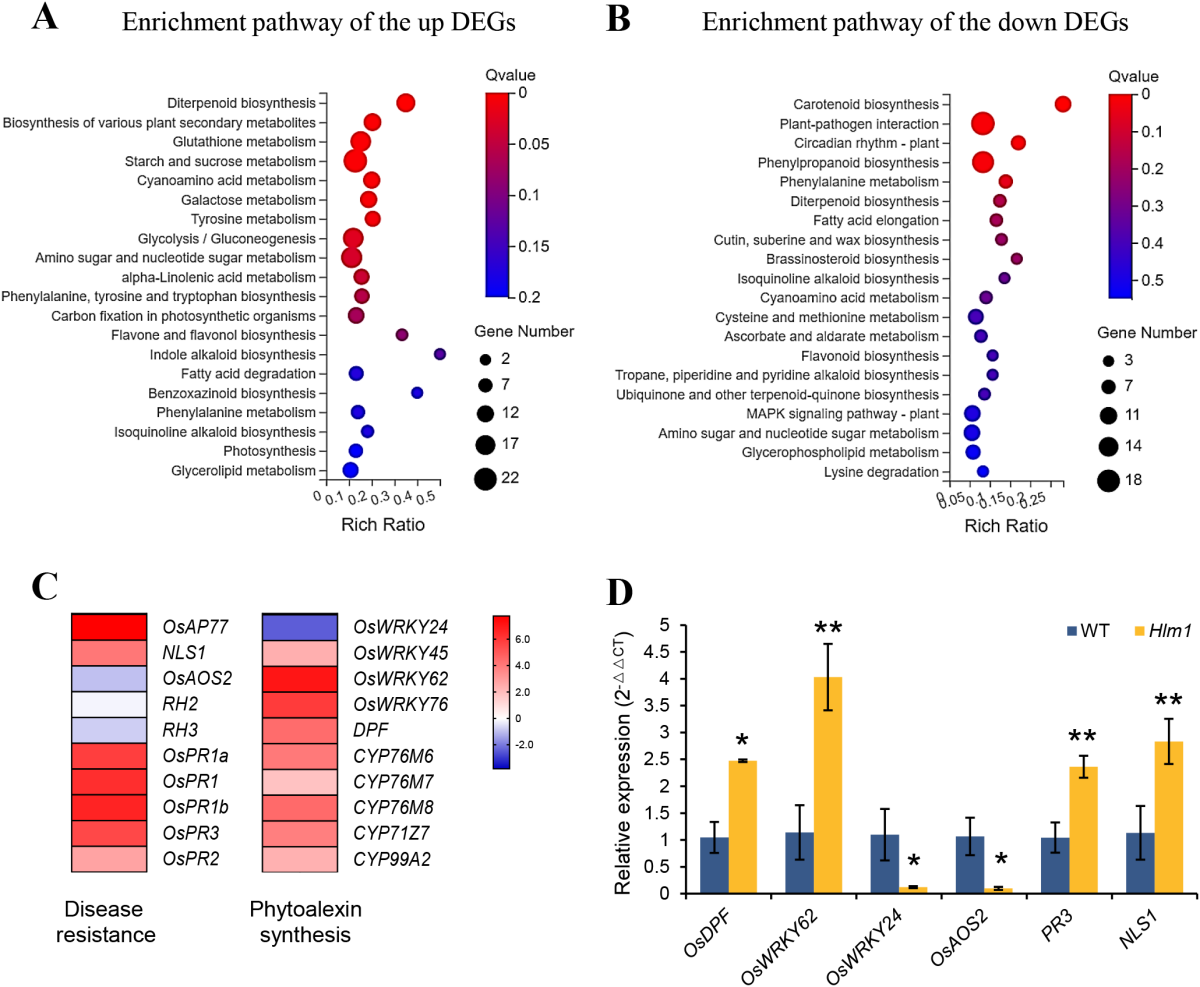
Enrichment pathways of the different expressed genes (DEGs) between the *Hlm1* mutant and WT. The KEGG pathway enrichment of the up-regulated DEGs **(A)** and down-regulated DEGs **(B)** between the *Hlm1* mutant and WT. The value on the right side of the column indicates the reliability of the enrichment degree of the pathways (*Q*-value). **(C)** The heat map of different expressed genes (DEGs) involved in responses to PCD and disease resistance. The DEGs between the *Hlm1* mutant and WT associated with disease resistance and diterpenoid phytoalexin synthesis were selected, the log values of the fold change of DEGs between the *Hlm1* mutant and WT were used for the display of heat map. **(D)** Verification of the relative expression levels of DEGs in WT and *Hlm1* mutant plants. Data are shown as means (three independent replicates) ± SD. Statistical analysis was performed with t-test. * represents significance differences exist at *p* < 0.05; ** represents significance differences exist at *p* < 0.01.

To further elucidate the signaling pathways of PCD and disease resistance in the *Hlm1* mutant, transcriptomes of the *Hlm1* mutant compared to WT were further analyzed by RNA-seq. Totally 2,805 differential expression genes (DEGs) were identified (fold change ≥ 2, adjusted *Q* < 0.05). Among them, 1,780 genes were up-regulated and 1025 genes were down-regulated in the *Hlm1* mutants when compared to WT (**Supplementary Table S1**). The Kyoto Encyclopedia of Genes and Genomes (KEGG) pathway enrichment analysis revealed that the down-regulated DEGs were preferentially involved in PCD and disease resistance such as plant-pathogen interaction (ko04626), phenylpropanoid biosynthesis (ko00940) and MAPK signaling pathway-plant (ko04016); while biosynthetic pathways associated with plant defense mechanisms, such as diterpenoid biosynthesis (ko00904) and biosynthesis of various plant secondary metabolites (ko00999) were enriched in the up-regulated DEGs (**Figures 6A, B**; **Supplementary Table S2**).

Notably, the plant-pathogen interaction signaling pathway and the MAPK signaling pathway-plant were the core regulatory networks of the plant immune system, and the activation of disease resistance genes relied on the integration and transmission of these pathways, such as *OsAP77*, *PR1, PR2* and *PR3* related to salicylic acid, and *OsAOS*2, *RH2* and *RH3* involved in jasmonic acid response, all of which were significantly regulated in the *Hlm1* mutant (**Figure 6C**). The diterpenoid phytoalexin synthesis pathway has been demonstrated to enhance plant disease resistance under biotic stress (Ahuja et al., 2012), and key components involved in this pathway, including the *OsWRKY45-OsWRKY62/OsWRKY76-DPF* regulatory module and a series of cytochrome P450 oxidases, were also found to be activated in the mutant (**Figure 6C**). More intriguingly, two lesion mimic genes named as *NLS1* and *DPF* respectively, which exhibited HR-like phenotype and led to constitutive activation of defense responses when ectopic expression in plants (Tang et al., 2011; Yamamura et al., 2015), were up-regulated in the *Hlm1* mutant (**Figure 6C**), potentially representing a key factor contributing to the HR phenotype observed in the mutant. The transcription levels of selected genes, including *DPF*, *OsWRKY62*, *OsWRKY24*, *OsAOS*2, *PR3* and *NLS1*, were verified via RT-qPCR, and the results were consistent with the RNA-seq data (**Figure 6D**). The results conclusively demonstrate that the observed PCD and enhanced disease resistance in the *Hlm1* mutant are mechanistically attributed to the signaling pathway-mediated activation of defense genes and concomitant promotion of diterpenoid phytoalexin biosynthesis.

## 4 Discussion

### 4.1 T-DNA insertion mutants contribute to functional genomic and have complex effects

Since the completion of rice whole genome sequencing, systematical identification of the biological functions of all genes has become the most challenging task in rice research. An effective way for analyzing gene function is to employ genetic mutations, such as mutants induced by T-DNA insertion or genome editing mediated knockout (Wei et al., 2013). Recently, with the rapid development of genome editing, knockout mutants have established a bridge between gene function and phenotype. However, knockout mutants are not always effective in functional study because of genetic redundancy and lethal mutation (Wang et al., 2013). Therefore, dominant mutations induced by the activation of T-DNA insertion play an irreplaceable role in functional research. In the present study, we obtained a lesion mimic mutant *Hlm1* from a transgenic line of Nipponbare, and proved that the phenotype of the *Hlm1* mutant is co-segregated with the T-DNA insertion and controlled by a single dominant gene locus (**Figures 1**, **2**). Subsequently, both flanking sequence analysis and map-based cloning located the target region in the same interval, in which the expression of three functional genes were significantly changed, including the decreased expression of *LOC_Os02g03880*, and the increased expression of *LOC_Os02g03890* and *LOC_Os02g03900* (**Figure 2**). Those results indicated that the effects of a single T-DNA insertion are not unique, but it can activate or inhibit the expression of adjacent genes simultaneously. Moreover, the effect of T-DNA insertion is not always the closer the stronger.

### 4.2 *OsNRAT1* is the candidate gene of the *Hlm1* mutant demonstrating lesion mimic phenotype

Since *Hlm1* is a dominant mutant, the strongly activated gene *LOC_Os02g03900* which encodes the Nramp aluminum transporter 1 (OsNRAT1) was further considered as the candidate gene of *Hlm1*. In *Arabidopsis*, which does not possess an *OsNRAT1* homolog, when ectopic expression of *OsNRAT1* gene in *Arabidopsis* significantly enhanced its tolerance to Al. More detailed analysis of these data demonstrated that there is a strong positive association between the level of *OsNRAT1* expression and Al tolerance (Li et al., 2014). Therefore, we subsequently tested the tolerance ability of the *Hlm1* mutant to Al, and the results confirmed that the *Hlm1* mutant truly acquired enhanced tolerance to different concentrations of Al (**Figure 3**). These results indicated that *LOC_Os02g03900* is the candidate gene of *Hlm1* and plays a key role in aluminum tolerance (Li et al., 2014). Subsequently, CRISPR/Cas9 mediated gene knockout of *OsNRAT1* was carried out in the *Hlm1* mutant, and the lesion mimic phenotype was significantly decreased in all of the knockout lines (**Figure 4**). The possible reasons for why the knockout of *OsNRAT1* genes by frameshift mutation still display slightly lesion mimic phenotype on the tip of leaves as follows. On the one hand, it may be that there are new ORFs in the genome after frameshift mutation, which transcribe new transcripts and play partial functions. On the other hand, it can’t be ruled out that it may be the result of the joint action of another activating expressing gene (*LOC_Os02g03890*), although its homologous gene has not been reported to contribute to lesion mimic phenotype. In the future, these possibilities will be verified through double knockout experiments or complementation assays. Hence, our results revealed a new function of the *OsNRAT1* gene in inducing lesion mimic.

### 4.3 OsNRAT1 regulating PCD and disease resistance might be through OsSPL1

**Figure 7:**
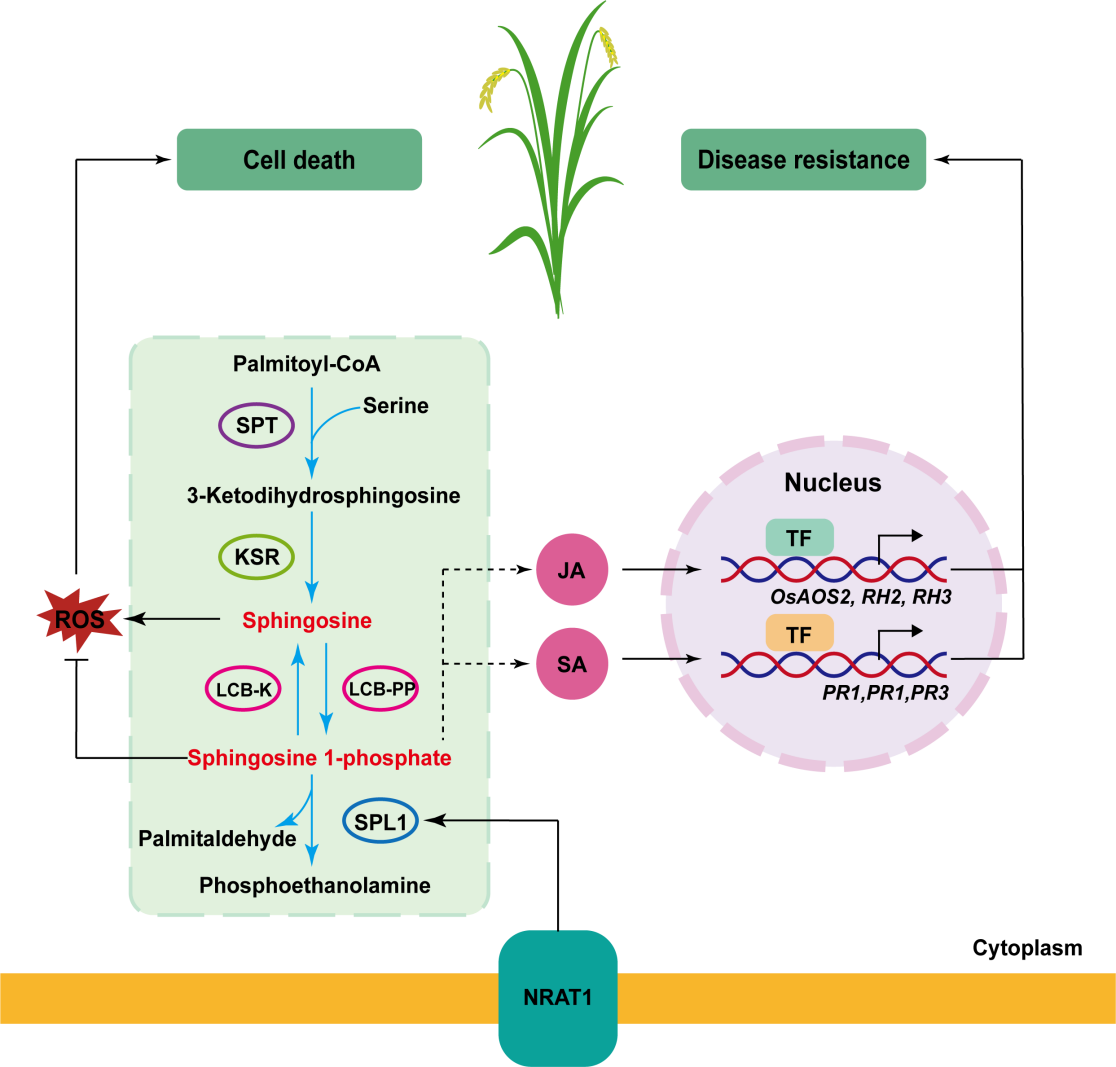
Signaling pathways of OsNRAT1 in programmed cell death and disease resistance. The OsNART1-OsSPL1 interaction modulates intracellular sphingosine-1-phosphate levels, where decreased S1P content leads to sphingosine accumulation and reactive oxygen species (ROS) burst, consequently triggering programmed cell death in rice; simultaneously, S1P enhances rice resistance to bacterial blight by activating salicylic acid (SA) or jasmonic acid (JA) pathways to promote the transcription of disease resistance-related genes via transcription factors. The green box outlines S1P biosynthesis and catabolic pathways, with blue arrows indicating the anabolic direction. Black arrows and lines with an end line indicate positive regulation and negative regulation, and the dash arrow represents indirect effects through unknown intermediate factors. SPT, serine palmitoyltransferase; KSR, 3-ketosphinganine reductase; LCB-K, long-chain base kinase; LCB-PP, long-chain base phosphate phosphatase; SPL1, sphingosine 1-phosphate lyase; SA, salicylic acid; JA, jasmonic acid; TF, transcription factor.

Studies have established lipid signaling as an integral component of the complex regulatory networks governing plant responses to diverse biotic and abiotic stresses (Wang et al., 2006; Munnik and Testerink, 2009). Sphingolipids, including sphingosine-1-phosphate (S1P) and ceramides, serve not only as essential membrane constituents but also as critical signaling mediators in stress responses. Enzymes involved in sphingolipid metabolism, play pivotal roles in plant defense mechanisms and PCD (Shi et al., 2007; Saucedo-García et al., 2011; Ternes et al., 2011). Y2H and BiFC proved that OsNRAT1 interacted with OsSPL1 by proteins pattern (**Figure 5**). Biochemical characterization reveals that *OsSPL1* encodes a functional sphingosine-1-phosphate lyase involved in S1P catabolism (van Veldhoven and Mannaerts, 1991), and *OsSPL1* negatively regulates disease resistance against pathogen infection while positively modulating PCD in rice, indicating its dual regulatory roles in immunity and cell death processes (Zhang et al., 2014). Moreover, *OsSPL1* may modulate the balance of salicylic acid (SA)-mediated defense pathways and jasmonic acid/ethylene (JA/ET) signaling pathways to activate defense responses upon pathogen attack (Zhang et al., 2014). RNA-seq analysis further revealed significant alterations in SA pathway-associated defense genes (*PR1, PR2, PR3*) and JA pathway-related genes (*OsAOS*2, *RH2*, *RH3*) in the *Hlm1* mutant (**Figure 6**). We therefore hypothesize that the elevated expression of *OsNRAT1* in the *Hlm1* mutant regulates OsSPL1, leading to altered levels of S1P which subsequently modulate both SA and JA signaling pathways, thereby activating plant defense responses against pathogens and resulting in the lesion-mimic phenotype (**Figure 7**). Naturally, the molecular mechanism by which OsNRAT1 interacts with OsSPL1 to regulate stress responses in rice could be further validated through quantitative analysis of S1P, salicylic acid, and jasmonic acid levels in both the *Hlm1* mutant and its corresponding knockout plants.

### 4.4 Up-regulating *OsNRAT1* activates the disease resistance and phytoalexin biosynthesis pathway

It is not rare to find that a single *LMM* gene contributed to multiple biological processes, such as *SPL11* negatively regulates programmed cell death and disease resistance by inhibiting the production of ROS, while positively regulates flowering via interaction with SPIN1 (Zeng et al., 2004; Vega-Sánchez et al., 2008). Here we reported that *OsNRAT1* not only functions in aluminum tolerance as previously characterized, but also confers resistance to rice bacterial blight (**Figure 1**). Recently, another metal transporter OsNRAMP1 was proved to regulate disease resistance by modulating ROS homeostasis (Chu et al., 2022). In our study, the burst of ROS was also found in the *Hlm1* mutant (**Figure 1**). Currently, the detailed mechanisms of *OsNRAT1* gene are not clear, the pathways of *OsNRAT1* involved in PCD and disease resistance are still confused. However, the transcriptomes of the *Hlm1* mutant provided some valuable insight. Through RNA-seq analysis, we demonstrated that OsNRAT1 enhances rice disease resistance primarily by regulating defense-related plant-pathogen interaction signaling pathways and MAPK signaling pathway to activate defense gene expression, while concurrently triggering the diterpenoid phytoalexin biosynthesis pathway to produce antimicrobial phytoalexins against pathogen invasion. Therefore, the molecular mechanism underlying of *OsNRAT1* gene will be helpful to deepen the understanding of the disease resistance mechanisms in rice.

## 5 Conclusions

In this study, we identified a dominant lesion mimic mutant *Hlm1* which enhanced disease resistance to rice bacterial blight, and confirmed that the lesion mimic phenotype of the *Hlm1* mutant was controlled by the ectopic expression of *OsNRAT1.* The primary mechanism involves OsNRAT1-mediated regulation of downstream programmed cell death (PCD)-associated and disease resistance-related gene expression through its interaction with OsSPL1 in both the cytoplasm and plasma membrane.

## Data Availability

The datasets presented in this study can be found in online repositories. The names of the repository/repositories and accession number(s) can be found below: BioProject, PRJNA1293528.
